# Relationships of Thyroid Hormones with Polychlorinated Biphenyls, Dioxins, Furans, and DDE in Adults

**DOI:** 10.1289/ehp.10179

**Published:** 2007-05-31

**Authors:** Mary E. Turyk, Henry A. Anderson, Victoria W. Persky

**Affiliations:** 1 Division of Epidemiology and Biostatistics, School of Public Health, University of Illinois at Chicago, Chicago, Illinois, USA; 2 Wisconsin Division of Public Health, Bureau of Environmental Health, Madison, Wisconsin, USA

**Keywords:** CDC, Centers for Disease Control and Prevention, DDE, dioxin, endocrine disruption, furan, National Health and Nutrition Examination Survey, NHANES, PCB, thyroid, thyroid-stimulating hormone, thyroxine, toxic equivalents, TSH

## Abstract

**Background:**

Thyroid hormone homeostasis can be disrupted by exposure to ubiquitous and bioaccumulative organochlorines such as polychlorinated biphenyls (PCBs) and polychlorinated dibenzo-*p*-dioxins (PCDDs). Whereas investigations of health effects have generally focused on human populations with relatively high exposures through occupation, accident, or high fish consumption, general population exposures may also carry risk.

**Methods:**

We studied associations of total thyroxine (T_4_) and thyroid-stimulating hormone (TSH) with PCBs, dioxin-like toxic equivalents (TEQs), and *p,p*′-diphenyldichloroethene (DDE) in adult participants without thyroid disease who participated in the 1999–2002 National Health and Nutrition Examination Survey, a cross-sectional survey examining a random sample representative of the U.S. population.

**Results:**

We found inverse associations of total T_4_ with exposure to TEQs in both sexes, with stronger associations in females. In women, mean T_4_ was 8.2 μg/dL, and levels were on average 0.75 μg/dL lower (95% confidence interval, 0.04–1.46) in women in the highest quintile of TEQ exposure compared with the lowest two quintiles. Effects were stronger in people > 60 years of age, with negative associations of T_4_ with PCBs and TEQs, and positive associations of TSH with PCBs and TEQs in older women, and a negative association of TSH with PCBs in older men.

**Conclusions:**

The data show a dose-dependent decrease in total T_4_ with exposure to TEQs at levels similar to those found in the general U.S. population. The effects were stronger in women. The results suggest that older adults, who have a high risk of thyroid disease, may be more at risk for disruption of thyroid hormone homeostasis by dioxin-like organochlorines than younger adults.

Polychlorinated biphenyls (PCBs), polychlorinated dibenzo-*p*-dioxins (PCDDs), polychlorinated dibenzofurans (PCDFs), and *p,p*′-diphenyldichloroethene (DDE) are widespread persistent environmental contaminants. Although human body burdens of these chemicals have been decreasing over time (Hagmar et al. 2006; Schecter et al. 2005), they remain detectable in most of the population due to their long half-life in the body (Geyer et al. 2002) and continued exposure primarily through the food supply (Needham et al. 2005).

Organochlorines have been associated with a number of health effects, including disruption of thyroid hormone homeostasis. Thyroid hormones are under control of the hypothalamo–pituitary–thyroid (HPT) axis. Reduction of circulating thyroxine (T_4_) is compensated for by release of thyroid-stimulating hormone (TSH) from the pituitary, which in turn stimulates the thyroid to produce more hormones. In animals, PCBs and dioxin-like compounds disrupt the HPT axis, decreasing thyroxine levels and causing inconsistent changes in TSH (Brouwer et al. 1990; Fisher et al. 2006; van Birgelen et al. 1992, 1995). Dioxin-like chemicals are thought to accomplish this through binding to the aryl hydrocarbon receptor (AhR), which induces uridine diphosphate glucuronosyltransferase enzymes, leading to increased glucuronidation and excretion of T_4_ (Fisher et al. 2006; van Birgelen et al. 1995). *Ortho*-substituted PCBs, which do not bind to the AhR, disrupt the HPT axis through other mechanisms which may include increased glucuronidation through non AhR pathways, displacement of T_4_ from the binding protein transthyretin, and direct effects on TSH release from the pituitary (Brouwer et al. 1990; Khan et al. 2002; van Birgelen et al. 1992, 1995).

The effects of organochlorines on thyroid hormone homeostasis have been studied in humans, but results have not been consistent. Most investigations of endocrine disruption by PCBs, PCDDs, PCDFs, and DDE in humans have focused on populations with higher exposures due to occupation or residence near areas contaminated by industry (Calvert et al. 1999; Langer et al. 1998, 2004; Osius et al. 1999; Ott et al. 1994; Pavuk et al. 2003; Persky et al. 2002; Schell et al. 2004; Triebig et al. 1998), accident (Murai et al. 1987), or fish consumption (Hagmar et al. 2001a, 2001b; Langer et al. 2007; Persky et al. 2001; Rylander et al. 2006; Turyk et al. 2006a). General population exposures have also been associated with thyroid disruption (Meeker et al. 2007; Takser et al. 2005), although studies have not usually been population based. A number of factors may be related to the inconsistent human findings, including different detection methods for biomarkers and endogenous hormones, varying overall exposure levels and concomitant chemical exposures, as well as differences in age, sex, nutritional status, comorbidities, and medication use.

In this study, we examined the effects of low-level general population organochlorine exposures on endogenous thyroid hormones in a population without reported thyroid disease. The National Health and Nutrition Examination Survey (NHANES) is a cross-sectional survey examining a random sample representative of the U.S. population [Centers for Disease Control and Prevention (CDC) 2007]. During the 1999–2000 and 2001–2002 survey rounds, PCB, PCDD, and PCDF congeners; DDE; total T_4_; and TSH were measured in approximately one-third of the NHANES sample. Data on individual organochlorine congeners have been presented in the Third National Report on Human Exposure to Environmental Chemicals (CDC 2005) and by Needham et al. (2005). This article focuses on the cross-sectional relationships of PCB, dioxin-like toxic equivalent (TEQ), and DDE body burdens with T_4_ and TSH serum levels in these two NHANES samples.

## Methods

### Participants

Data from NHANES survey cycles conducted in 1999–2000 and 2001–2002 were obtained online (CDC 2007). During these two data collection cycles, total T_4_, TSH, DDE, and PCB, PCDD, and PCDF congeners were measured in serum sampled from one-third of the participants. This subsample is also a nationally representative sample of the U.S. population (CDC 2007). Participants < 20 years of age were not tested for PCDD and PCDF congener data during the 2001–2002 cycle; as a result, they were excluded from the analysis. After exclusion of participants for which we did not have both organochlorine and thyroid hormone measurements and those who were diagnosed with current thyroid disease or who used thyroid medication (*n* = 150; 36 men and 114 women), a total of 995 participants for the 1999–2000 cycle and 1,450 participants for the 2001–2002 cycle were available for analysis of the associations of thyroid hormones with organochlorine body burdens. We were not able to examine associations of diagnosed hypothyroidism with organochlorines because the survey questions relating to thyroid conditions did not distinguish between different types of thyroid diseases.

### Thyroid hormones

Total T_4_ (micrograms per deciliter) and TSH (international units per liter) were measured in serum by two different laboratories. Sera collected during the 1999–2000 cycle and part of the 2001–2002 cycle were measured by the Coulston Foundation (Alamogordo, NM), whereas the remainder of samples from the 2001–2002 cycle were tested at Collaborative Laboratory Services (Ottumwa, IA). The National Center for Health Statistics evaluated the 2001–2002 TSH and T_4_ data sets from the two laboratories and determined that the variables were comparable across the 2 years (Blount et al. 2006). Total T_4_ was measured on a Hitachi 704 chemistry analyzer (Coulston Foundation) and by a paramagnetic particle, chemiluminescent, competitive binding enzyme immunoassay (Collaborative Laboratory Services) (CDC 2007e). TSH was measured by an IMx ultrasensitive hTSH II microparticle enzyme immunoassay (Coulston Foundation) and by a two-site, paramagnetic particle and chemiluminescent immunoassay (Collaborative Laboratory Services) (CDC 2007d). Both laboratories reported a reference range of 5.4–12.8 μg/dL for total T_4_. Reference ranges for TSH were 0.47–5.0 IU/L for the Coulston Foundation and 0.24–5.4 IU/L for Collaborative Laboratory Services.

### PCB, PCDD, PCDF, and DDE measurements

Organochlorines were measured in serum by high-resolution gas chromatography/isotope-dilution high-resolution mass spectrometry (Organic Toxicology Branch, National Center for Environmental Health, CDC, Atlanta, GA) (CDC 2007b, 2007c). We created a variable for total PCBs (∑PCBs) by summing individual PCB congeners. TEQs were calculated for each PCDD, PCDF, coplanar PCB, and mono-*ortho* PCB congener by multiplying the toxic equivalency factor by the congener concentration in picograms per gram (Van den Berg et al. 2006) and then summing the values to calculate total TEQs (∑TEQs). For congeners with results below the limit of detection (LOD), the CDC imputed the value for the congener as the LOD for that specific congener divided by the square root of 2. The LOD varied for each participant, as it was dependent on the volume of the sample submitted for analysis. In the first sampling cycle (1999–2000), fewer congeners were measured and more individual results were below the LOD, compared with the second cycle (Table 1). Only congeners that had > 10% of results > LOD were included in the ∑PCBs and ∑TEQs; therefore, the specific congeners in the ∑PCBs and ∑TEQs differed in the two sampling cycles (Table 1). When results for more than one congener were not reported by the CDC for a participant, the participant was coded as missing for ∑PCBs or ∑TEQs.

### Covariates

We considered medications that can alter hormone homeostasis (Meier and Burger 2005) to be potential effect modifiers or confounders of the associations of organochlorines on thyroid hormones. Medications were identified in the prescription drug medication and the analgesics/pain reliever questionnaires, and included estrogens and/or progesterone, other steroid hormones (androgens, adrenal corticosteroids, tamoxifen, raloxifene, and pituitary hormones), non-steroidal anti-inflammatory drugs (NSAIDs), furosemide, beta-blockers, blood glucose regulators, and other medications thought to affect thyroid hormones (amidoarone, carbamazepine, chlorpropamide, carbidopa/levodopa, heparin, interferon, lithium, phenytoin, phenobarbital, and sulfasalazine). Because estrogen alters total thyroxine-binding globulin concentrations, we also considered current pregnancy and menopausal status to be potential confounders or effect modifiers. Smoking can affect thyroid hormone levels through the metabolism of cyanide in smoke to thiocyanate, a potent inhibitor of iodide transport (Meier and Burger 2005). We used serum cotinine levels to estimate tobacco smoke exposure (Organic Analytical Toxicants Branch, National Center for Environmental Health, CDC). Serum lipids are generally associated with serum organochlorine concentrations because of partitioning, and they also are often increased in hypothyroidism. We calculated total serum lipids using the formula given in the NHANES laboratory manual for dioxins (CDC 2007c):


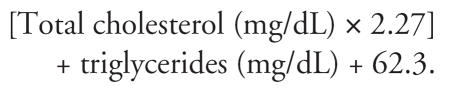


Age (years), sex, race (Mexican American, African American, Caucasian, or other), and body mass index (BMI) were also included as covariates. BMI was calculated from height and weight measured during the NHANES examination (weight in kilograms ÷ height in meters squared). For 85 of the 91 participants who were missing measured BMI, we imputed BMI using self-reported weight and height. In participants with both measures, the correlation between self reported and measured BMI was > *r* = 0.9.

### Statistical analyses

NHANES uses a complex sampling design that requires the use of sample weights to adjust for the unequal probability of selection into the survey and to adjust for the possible bias resulting from non-response; weights are poststratified to U.S. Census Bureau estimates of the U.S. population. Data management and analyses were performed with SAS 9.1 (SAS Institute Inc., Cary, NC), using sample weights for the individual 2-year cycles or 4-year combined cycles, as appropriate, and calculating variances that accounted for the complex survey design.

Because the data were not normally distributed, we used natural log (ln)-transformations of TSH, ∑PCBs, ∑TEQs, DDE, BMI, and cotinine for analysis. For univariate analyses, we calculated means with SAS PROC SURVEY-MEANS, using the domain command to estimate means in subpopulations; differences between groups were assessed with SAS PROC SURVEYREG. Categorical data was evaluated with SAS PROC SURVEYFREQ, and differences between groups were tested using SAS PROC SURVEYLOGISTIC.

Associations of thyroid hormones with ∑PCBs, ∑TEQs, and DDE were modeled using PROC SURVEYREG. SAS does not allow for subpopulation analyses in PROC SURVEYREG; therefore, we used Stata 6.0 (StataCorp LP, College Station, TX) to calculate the variances for the subpopulation models. Stata uses a variance estimator that accurately measures the sample-to-sample variability of the subpopulation estimates for the survey design used to collect the data (StataCorp 2005). Because the ∑PCBs and ∑TEQs were significantly higher in the second sampling cycle (Tables 1 and 2), associations were tested for each sampling cycle individually, using continuous predictor variables. We could not directly combine sampling cycles for analysis because fewer congeners were summed and more individual results were < LOD in the first sampling cycle (Table 1). To combine data from both cycles for analysis, we assumed that the true exposure levels of the U.S. population did not change substantially between 1999–2000 and 2001–2002, and, therefore, that the ranks of the exposure measurements for each cycle should be comparable. However, ranking should be more valid for ordering participants with high rather than low exposures, because most of the ∑PCBs and ∑TEQs that were composed of a large proportion of congeners < LOD fell into the lower ranks. Thus, data from both sampling cycles were combined by ranking the ∑PCB, ∑TEQ, and DDE levels into quintiles separately for each cycle, merging the data from both cycles, and pooling the lowest two quintiles. Dose–response models were estimated using indicator variables for quintiles 3, 4, and 5, with quintiles 1 and 2 combined as the reference category, or the ordinal variable (quintile 1–2, 3, 4, 5), to test for a trend over the categories. For all regression analyses we used sample weights (wet weight) of PCB, PCDD, and PCDF congeners rather than lipid-standardized measurements, and we included serum lipids as a covariate (Schisterman et al. 2005).

Organochlorines and thyroid hormones were associated with age and with many of the potential covariates we identified prior to the analysis. We therefore evaluated relationships of the covariates with exposure and outcome variables after controlling for age. TSH was negatively associated with cotinine and post-menopausal status, and positively associated with BMI and use of other medications (*p* < 0.05). T_4_ was negatively associated with lipids, cotinine, and furosamide use, and positively associated with estrogen/progesterone use and pregnancy (*p* < 0.05). At least one exposure was positively associated (*p* < 0.05) with lipids, BMI, diabetes medication use, estrogen/progesterone use, beta-blocker use, and postmenopausal status, and negatively associated with steroid hormone use, other medication use, and serum cotinine. Regression models were individually adjusted for serum lipids, age, log BMI, race, log cotinine and use of NSAIDs, furosemide, beta-blockers, blood glucose regulators, other medications, and, for women, completion of menopause. Analyses of the combined study cycles were also adjusted for cycle. Participants taking estrogen and/or progesterone (*n* = 201) or other steroid hormones (*n* = 70; 40 women and 30 men), and pregnant participants (*n* = 163) were excluded from the analyses because potential modification of the effects of organochlorines on thyroid hormones by estrogen and/or progesterone medications was noted in stratified analyses.

## Results

Demographic information, medication use, and thyroid hormone levels for participants without thyroid disease are shown in Table 3. Total T_4_ was higher in females than males, but TSH did not differ by sex (Table 3). T_4_ was higher and TSH was lower in the 2001–2002 cycle compared with the 1999–2000 cycle (*p* < 0.05; data not shown). BMI, lipids, TSH, percent with TSH > 5.0 IU/L, and use of medications (estrogens and/or progesterones, other steroid hormones, beta-blockers, NSAIDs, and blood glucose regulators) increased with age, whereas cotinine exposure decreased with age (*p* < 0.05; data not shown).

∑PCBs, ∑TEQs, and DDE were positively associated with age in males and females (*p* < 0.05), but mean levels did not differ significantly by sex (*p* > 0.05; data not shown). Correlations among organochlorines were positive, with the strongest associations between ∑PCBs and ∑TEQs (range, *r* = 0.44–0.82). The main congeners contributing to ∑PCBs were PCBs 138, 153, and 180, which comprised 54% and 41% of the ∑PCBs in the first and second cycles, respectively, with these individual congeners highly correlated with ∑PCBs (*r* = 0.90–0.99). Approximately 76% of ∑TEQs were from the congeners 1,2,3,7,8-pentaCDD, 1,2,3,6,7,8-hexaCDD, 2,3,4,7,8-pentaCDF, and PCB-126 in cycle 1 and from these four congeners plus 2,3,7,8-tetraCDD in cycle 2. TEQs for these congeners were significantly associated with ∑TEQs (*r* = 0.71–0.89), with stronger associations in the second cycle than the first cycle. Because both ∑PCBs and ∑TEQs were higher in the second cycle than in the first cycle (Table 2), we first modeled the relationships of these organochlorines with thyroid hormones separately for each sampling cycle (Tables 4 and 5).

Total T_4_ was negatively associated with total ∑TEQs in men and women, with stronger associations of T_4_ with ∑TEQs for both men and women in the second sampling cycle compared with the first cycle (Tables 4 and 5). Results, however, were statistically significant only in older women and men; in older men the results were inconsistent and were statistically significant only in the second cycle with further adjustment for ∑PCB and DDE levels. In women, TSH was positively associated with ∑TEQs, with a statistically significant association only in older women in the second sampling cycle. In men, associations of TSH with ∑TEQs were generally negative, but not significant.

Associations of ∑PCBs with T_4_ and TSH were inconsistent in women. In older women, ∑ PCBs were negatively associated with T_4_ and positively associated with TSH, with statistically significant associations only in the second cycle (Table 4). In men, TSH was negatively associated with ∑PCBs; associations were statistically significant in older men during the first cycle and during the second cycle with further adjustment for ∑TEQs and DDE. Associations of T_4_ with ∑PCBs in men were inconsistent and were not statistically significance (Table 5).

T_4_ was positively associated with DDE in all women and in younger women, with a statistically significant association only in the first cycle in younger women. In older women, the direction of the association differed by cycle (Table 4). In men, T_4_ was negatively, but not significantly, associated with DDE; again the direction of the association differed in older participants (Table 5). Associations of TSH with DDE were inconsistent and not significant.

**Figure 1:**
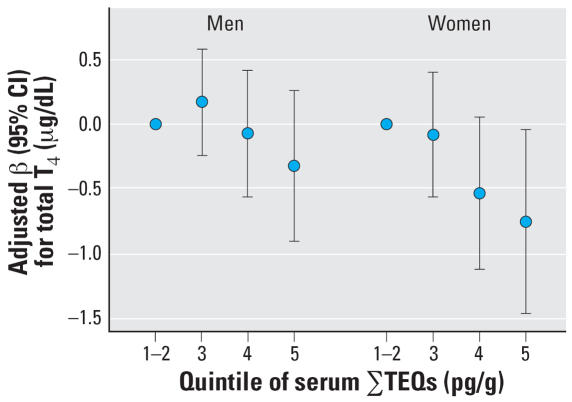
Associations [β (95% CI)] of total T_4_ with ∑TEQs in participants from both the 1999–2000 and the 2001–2002 NHANES cycles. ∑TEQs were ranked into quintiles within each individual cycle, the cycles were merged, and the lowest two quintiles were combined for analysis. Models were adjusted for survey design, sample weights, study cycle, total serum lipids, log BMI, race, age, log serum cotinine, medication use (furosamide, NSAIDs, beta-blockers, blood glucose regulators, and other medications), and menopause status (women only). Adjusted beta coefficients (95% CIs) for trends across quintiles were −0.09 (−0.28 to 0.10) for men (*p* = 0.28, *n* = 899) and −0.25 (−0.48 to −0.02) for women (*p* = 0.03, *n* = 696). With further control for DDE, trend across quintiles remained significant for women (*p* < 0.05). With further control for ∑PCBs, results were of borderline significance for women (*p* = 0.07).

Data from both sampling cycles were combined by ranking the exposure measurements into quintiles for each individual cycle, merging the data from both cycles, and combining the lowest two quintiles. We found a dose response for the associations of ∑TEQs with T_4_ for women and men, with a significant trend for the dose only for women (Figure 1). The decrease in total T_4_ with an increase in one quintile of ∑TEQs was 0.25 μg/dL [95% confidence interval (CI), 0.02–0.48] for women. The T_4_ decrease was 0.75 μg/dL (95% CI, 0.04–1.46) for women in the highest TEQ quintile compared with the lowest. The association for women remained significant or of borderline significance with further adjustment for quintile ∑PCBs or quintile DDE. No other significant associations were found for the combined data cycles for T_4_ with PCBs or DDE or for TSH with any exposure in either men or women.

We repeated the analyses using a different method to calculate ∑PCBs and ∑TEQs. Only congeners detectable in > 50% of participants were included in the ∑PCBs and ∑TEQs (Table 1). Results were generally similar for the analyses of data from the individual cycles, except T_4_ was not significantly associated with ∑TEQs in older women in the first cycle, and T_4_ became significantly associated with ∑TEQs in older men in the second cycle (data not shown). In the combined cycle analysis, the association of ∑TEQs with T_4_ was slightly weaker in women and slightly stronger in men (0.05 < *p* < 0.15 for both; data not shown). In older men, the association of ∑PCBs with TSH in the first cycle did not remain significant (data not shown).

Because these data are a sample from the general population, we would expect that some participants might have unusually high contaminant exposures due to high sport fish consumption or occupation and abnormal thyroid hormone levels because of undiagnosed thyroid disease. To determine if model results were affected by extreme values, we excluded participants with exposure values more than three interquartile ranges above the 75th percentile (∑PCBs > 7 ng/g, *n* = 25; ∑TEQs > 0.62 pg/g, *n* = 20; DDE > 30 ng/g, *n* = 78) and participants with very high TSH (42.7, 44.0, 81.9, 234.6 IU/L) and T_4_ levels (27 μg/dL). Significant relationships between thyroid hormones and ∑TEQs remained. For men > 60 years of age, the negative association of ∑PCBs with TSH became significant in the second sampling cycle, but did not remain significant in the first sampling cycle; for women > 60 years of age in the second sampling cycle, the positive association of ∑PCBs with TSH did not remain significant (data not shown).

## Discussion

In the adult participants of NHANES from 1999 to 2002, total T_4_ was negatively associated with serum dioxin-like TEQs in a dose-dependent fashion, with stronger associations in women than men. Associations of organochlorines with thyroid hormones were stronger in participants > 60 years of age, with lower T_4_ and higher TSH with both PCB and TEQ exposure in women, and lower TSH with PCB exposure in men. With further adjustment for multiple exposures, the negative associations of T_4_ with TEQs generally remained significant or borderline significant.

Overall, results of human studies on the effects of PCBs, PCDDs, and DDE on thyroid hormones have been inconsistent. However, a variety of factors may be related to the inconsistent findings, the most important of which may include small numbers of participants, varying overall exposure levels, use of various detection methods for biomarkers and endogenous hormones, and differing age, sex, and unmeasured exposures to chemicals affecting hormone homeostasis.

In three studies with high exposures, dioxin-like chemicals have been associated with increased thyroid hormones. Occupational exposures to dioxin-like compounds were associated with increased levels of free T_4_ (mean 220 pg/g lipid TEQ; Calvert et al. 1999), as well as total T_4_ and thyroxine-binding globulin (range, < 1–533 pg/g lipid TEQ; Ott et al. 1994). Exposure to PCBs and PCDFs in the Yusho outbreak was associated with increased total triiodothyroxine (T_3_) and T_4_, but not TSH, 16 years after exposure (Murai et al. 1987), with a mean of 222.4 pg/g lipid TEQ 30 years after exposure (Nagayama et al. 2001). Results have varied more for lower exposures: with no association with total T_4_ or TSH in metal recyclers (mean, 42 pg/g lipid TEQ; Treibig et al. 1998); increased TSH but no change in total T_4_ in Vietnam veterans (mean, 45.7 pg/g lipid TEQ; Pavuk et al. 2003), decreased TSH but no association with total T_3_, total T_4_ or free T_4_ in male fish consumers (range, 11–105 pg/g lipid TEQ; Turyk et al. 2006a), and decreased total T_3_ and total T_4_, but no change in TSH and free T_4_, in pregnant women (mean, 74.9 pg/g lipid TEQ in breast milk; Koopman-Esseboom et al. 1994). The differential effects of dioxins on thyroid hormone homeostasis that appear to be related to exposure levels could potentially be attributed to down-regulation of the AhR after large exposures, such as noted after the Seveso (Italy) accident (Landi et al. 2003). In the present study, with an average TEQ exposure of 12–18 pg/g lipid, we observed decreased T_4_ with dioxin-like exposure, in agreement with Koopman-Esseboom et al. (1994).

Most studies of exposure to PCBs have found inverse associations with T_4_. A negative association with one or more thyroid hormones and positive associations with TSH have been found with measures of PCB exposure in children living near PCB-contaminated sites (Osius et al. 1999; Schell et al. 2004). Langer et al. (1998) reported that PCB production workers and controls from a less-polluted area had similar levels of total T_4_ and TSH, but a later study of adults from a heavily polluted area demonstrated positive relationships of PCBs with free T_4_ and free T_3_ (Langer et al. 2004). Male capacitor manufacturing employees with exposure to PCBs and chlorinated naphthalene had decreased TSH and no change in total T_4_ (Persky et al. 2002). In frequent fish consumers, an inverse association of PCB-153 was found with total T_3_ among women (Hagmar et al. 2001b) but not men (Hagmar et al. 2001a; Rylander et al. 2006). In a group of frequent Great Lakes fish consumers, Persky et al. (2001) found inverse associations of PCB levels with total T_4_ in men and women and with free T_4_ in women; in a different subgroup of participants from the same study, inverse associations of PCBs were found with total T_3_, total T_4_, and TSH in men (Turyk et al. 2006a). Much smaller effects of PCBs on thyroid hormones were noted in New York anglers (Bloom et al. 2003) and in a population in Spain (Sala et al. 2001). Negative associations were found for total T_3_ with low-level exposure to PCBs in pregnant women (Takser et al. 2005); negative associations were also found in men (Meeker et al. 2007), but only after controlling for DDE. In the present study we saw no effect of PCBs on thyroid hormones in the NHANES cohort as a whole, although we did find decreased T_4_ and increased TSH in older women and decreased TSH in older men. It is possible that there were effects on unmeasured thyroid hormones, such as free T_4_ or total T_3_, or that levels of PCBs were too low to affect thyroid homeostasis.

Few investigations have examined associations of DDE with thyroid hormones. No associations were found for DDE with thyroid hormones in male or female fish consumers (Hagmar et al. 2001a; Persky et al. 2001; Turyk et al. 2006a); a positive association was found with TSH in male fish consumers (Rylander et al. 2006); a negative association was found with total T_3_ in pregnant women with low levels of exposure (Takser et al. 2005); and positive associations were found with total T_3_ and free T_4_ in men with low exposure (Meeker et al. 2007). In the present study, we did not find any significant associations of DDE with thyroid hormones when both sampling cycles were combined, although T_4_ was positively associated with DDE in younger women, but only in the first sampling cycle. In older participants, associations in the first and second cycles were inconsistent.

The HPT axis normally responds to decreases in free T_4_ with increased production of TSH. In women > 60 years of age, we found that PCBs and TEQs were negatively associated with T_4_ and positively associated with TSH, which is consistent with a normal pituitary response to decreased T_4_ levels. Elevated TSH, even within the high-normal reference range, may be a marker for increased risk of hypothyroidism (Vanderpump 2005). In adults living in areas with sufficient iodide intake, the most common cause of hypothyroidism is autoimmune disease. Markers of autoimmune disease, such as anti-thyroperoxidase antibodies and thyroid hypoechogenicity, have been associated with PCB exposure (Langer et al. 2004, 2007). Our observation of decreased T_4_ and increased TSH in older women with higher exposure to dioxin-like TEQs or PCBs is intriguing because this population group has the highest risk of hypothyroidism, reaching an annual incidence rate of > 13/1,000 in women 75–80 years of age (Vanderpump 2005). The NHANES data set did not provide sufficient information on diagnosis of hypothyroidism to allow us to study the effects of organochlorine exposure on the prevalence of hypothyroidism. Overall, the decreases in T_4_ noted in this analysis may or may not be significant on an individual level, but they could substantially contribute to disease burden in the population.

Associations of organochlorines with thyroid hormones in the present study were stronger in females than in males, similar to results in studies of fish consumers (Hagmar et al. 2001a, 2001b; Persky et al. 2001), in children 7–10 years of age (Osius et al. 1999), and in infants (Wang et al. 2005). The stronger effects of organochlorines, particularly in older females, could be related to a number of age and/or sex-associated factors, including hormonal environment, organochlorine exposure and metabolism, and risk of developing preclinical and clinical thyroid disease. In these NHANES participants, PCBs and dioxin-like congeners differed by sex, with females having greater levels of dioxin-like congeners than males (Needham et al. 2005), which may be related to differential metabolism or elimination influenced by body fat or hormonal factors (Geyer et al. 2002).

Levels of PCBs, TEQs, and DDE in the NHANES participants in the 2001–2002 cycle were similar to those found in infrequent sport-fish consumers (Turyk et al. 2006b) and to age-specific PCDD, PCDF, and coplanar PCB TEQs in various U.S. populations (Patterson et al. 2004). PCBs and TEQs were significantly lower in participants during the 1999–2000 cycle than the 2001–2002 cycle, which limited our ability to draw conclusions about effects of these organochlorines on hormones for 1999–2000. Misclassification is more likely in the participants with lower levels of exposure because of the larger number of results < LOD, caused in part by laboratory limitations related to small serum volumes (Needham et al. 2005). We examined data from both cycles simultaneously to increase our power to detect sex-specific associations. To this end, we ranked both cycles into quintiles, merged them, and pooled the lowest two quintiles for analysis; although some residual misclassification may be present in this analysis, the results for TEQs and T_4_ are consistent with those found in the analyses of the second cycle alone. The inconsistencies in associations of DDE with thyroid hormones cannot be explained by differences in exposure levels by study cycle. An additional source of measurement error could come from the change in the laboratory performing the hormone tests during the second cycle, although the CDC has determined that values for TSH and T_4_ are comparable across the second cycle (Blount et al. 2006). In addition, any misclassification of the hormone levels in the second cycle should be nondifferential with regard to exposure and thus would be more likely to weaken associations in the second cycle. In fact, we saw stronger organochlorine–hormone associations in the second cycle compared with the first.

The cross-sectional design of the present study limits our ability to evaluate the temporal association of organochlorine exposure with thyroid hormone changes, but generally concentrations of organochlorines reflect long-term exposures with many congeners, particularly the more highly chlorinated congeners, persisting for years within the body (Geyer et al. 2002). We adjusted for many biological factors that could influence the relationship between thyroid hormones and organochlorines, but thyroid hormones affect several aspects of metabolism; thus, there may be other factors related to both serum levels of organochlorines and thyroid hormones for which we have not controlled. Additional hormone measurements, such as T_3_, free T_4_, and thyroxine-binding globulin, might have helped to elucidate mechanisms related to the associations we found between TEQs and thyroid hormones. In addition, the decreases in T_4_ could be associated with other unmeasured exposures, such as polybrominated biphenyl ethers or PCB metabolites, which are associated with the measured organochlorines. Although our main findings generally remained significant or borderline significant after adjustment for other measured organochlorines, evaluation of the effects of multiple exposures can be imprecise because of strong associations among exposures. Finally, the results for the subpopulation analyses should be viewed with caution in consideration of the sample size, which may be too small to produce reliable estimates using population-based statistical methodology.

In spite of the limitations and issues related to sample analysis for organochlorines and thyroid hormones described above, the present study has a number of strengths, including generally similar trends for results in both sampling cycles for the primary findings, large number of participants, population-based sampling design, and consistency with results of toxicologic studies in animals. Despite the fact that decreases of PCBs and/or PCDDs over time have been noted in cross-sectional and longitudinal studies (Hagmar et al. 2006; Schecter et al. 2005), the U.S. population continues to be exposed to low levels of these persistent chemicals, primarily through a dietary route. The data show a dose-dependent decrease in total T_4_ with exposure to dioxin-like TEQs, with an average decrease of 0.75 μg/dL, or 9% of average T_4_ levels, in the highest quintile compared with the lowest quintile in women, and suggests that older adults, who have a high risk of thyroid disease, may be more at risk for disruption of thyroid hormone homeostasis by organochlorines than younger adults.

## Figures and Tables

**Table 1:** Serum organochlorines in 1999–2002 NHANES participants without thyroid disease.

	Percent of participants with results < LOD		Median concentration (pg/g)	
Organochlorine	1999–2000 cycle	2001–2002 cycle	1999–2000 cycle	2001–2002 cycle
PCB-66^a^	NA	89	—	NC
PCB-74^a^	47	33	41	50
PCB-99^a^	59	38	NC	40
PCB-105^a^	86	79	NC	NC
PCB-118^a^,^b^	44	27	48	59
PCB-126^b^	36	12	0.12	0.16
PCB-138^a^	49	7	115	164
PCB-146^a^	66	53	NC	NC
PCB-153^a^	42	4	200	234
PCB-156^a^,^b^	58	46	NC	34
PCB-169^b^	36	12	0.09	0.13
PCB-170^a^	44	25	62	74
PCB-172^a^	NA	84	—	NC
PCB-177^a^	NA	83	—	NC
PCB-178^a^	87	81	NC	NC
PCB-180^a^	38	11	152	180
PCB-183^a^	80	70	NC	NC
PCB-187^a^	43	34	45	51
PCB-194^a^	NA	39	—	46
PCB-196^a^	NA	46	—	34
PCB-201^a^	NA	42	—	38
PCB-206^a^	NA	89	—	NC
2,3,7,8-TetraCDD^b^	NA	88	—	NC
1,2,3,7,8-PentaCDD^b^	83	67	NC	NC
1,2,3,4,7,8-HexaCDD^b^	NA	67	—	NC
1,2,3,6,7,8-HexaCDD^b^	52	7	NC	0.25
1,2,3,7,8,9-HexaCDD^b^	82	60	NC	NC
1,2,3,4,6,7,8-HeptaCDD^b^	28	1	0.25	0.28
1,2,3,4,6,7,8,9-OctaCDD^b^	22	19	1.78	2.33
2,3,4,7,8-PentaCDF^b^	47	35	0.03	0.03
1,2,3,4,7,8-HexaCDF^b^	51	18	NC	0.03
1,2,3,6,7,8-HexaCDF^b^	69	31	NC	0.03
2,3,4,6,7,8-HexaCDF^b^	NA	89	—	NC
1,2,3,4,6,7,8-HeptaCDF^b^	45	10	0.04	0.06
∑PCBs^c^	32	3	—	—
∑TEQs^c^	8	0	—	—

Abbreviations: —, not calculated because the congener was not measured; CDD, chlorodibenzo-*p*-dioxin; CDF, chlorodibenzofuran; NA, not tested or > 90% of results < LOD; NC, not calculated because > 50% of samples < LOD.

a Congener included in ∑PCBs.

b Congener included in ∑TEQ.

c Percent of participants with all congeners in ∑PCBs or ∑TEQs < LOD.

**Table 2:** Serum ∑PCBs, ∑TEQs, and DDE levels in 1999–2002 NHANES participants without thyroid disease.

	1999–2000 cycle			2001–2002 cycle		
Organochlorine	No.	GM^a^	95% CI	No.	GM^a^	95% CI
∑PCBs (ng/g)	945	0.86*	0.81–0.92	1,406	1.27*	1.20–1.35
∑PCBs (ng/g lipid)	945	139.8*	132.1–147.9	1,406	200.3*	189.3–212.1
∑TEQs (pg/g)	877	0.08*	0.07–0.08	1,107	0.12*	0.11–0.13
∑TEQs (pg/g lipid)	877	12.3*	11.6–13.0	1,107	18.2*	16.6–19.9
DDE (ng/g)	986	1.82	1.53–2.17	1,443	2.12	1.91–2.35
DDE (ng/g lipid)	986	293.0	248.0–346.1	1,443	337.0	304.3–373.1

GM, geometric mean.

a All estimates were adjusted for survey design and sample weights.

* Significantly different by study cycle (*p* < 0.05).

**Table 3:** Characteristics of the 1999–2002 NHANES participants without thyroid disease.^a^

	Males		Females	
Characteristic	Estimate^b^	95% CI	Estimate^b^	95% CI
No.	1,166		1,279	
Ethnicity (%)*				
Caucasian	72.5	67.4–77.7	68.8	63.9–73.6
African American	9.4	6.6–12.1	11.5	7.9–15.1
Mexican American	8.5	5.9–11.1	7.3	5.1–9.5
Other/mixed	9.6	5.5–13.6	12.4	8.6–16.3
Age [mean (years)]	44.9	43.6–46.3	45.9	44.6–47.1
BMI [geometric mean (kg/m^2^)]	27.3	26.9–27.8	27.0	26.5–27.5
Total serum lipids [mean (mg/dL)]	683*	660–707	652*	639–665
Cotinine [geometric mean (ng/mL)]	1.2*	0.8–1.8	0.4*	0.3–0.5
Completed menopause (%)	NA		45.5	41.9–49.1
Pregnant (%)	NA		3.7	2.6–4.7
Medication use in the past month (%)				
Estrogen and/or progesterone	NA		21.3	16.9–25.7
Other steroid hormones	2.3	1.1–3.4	3.2	2.3–4.1
Furosamide	2.2	1.1–3.4	2.7	1.7–3.8
Beta-blockers	6.7	4.0–9.5	5.6	4.3–6.9
NSAIDs	25.9	22.0–29.8	26.4	22.0–30.8
Blood glucose regulators	5.6	3.9–7.2	4.4	3.0–5.9
Other drugs^c^	0.8	0.3–1.3	1.0	0.3–1.7
Thyroid hormones				
T_4_ [mean (μg/dL)]	7.5*	7.3–7.7	8.2*	8.0–8.5
T_4_ [< 5.4 μg/dL (%)]	8.0*	5.3–10.6	3.2*	1.5–5.0
T_4_ [> 12.8 μg/dL (%)]	0.1*	0.0–0.2	1.5*	0.7–2.3
TSH [geometric mean (IU/L)]	1.44	1.39–1.50	1.46	1.39–1.53
TSH [< 0.47 IU/L (%)]	2.9	1.5–4.2	3.8	2.7–5.0
TSH [> 5.0 IU/L (%)]	2.4	1.4–3.5	2.3	1.4–3.2

NA, not applicable. Data were missing for cotinine (*n* = 21), BMI (*n* = 4), TSH (*n* = 3), pregnancy (*n* = 10), and completion of menopause (*n* = 25).

a A total of 36 men and 114 women with thyroid disease (reported current thyroid disease or taking thyroid medications) were excluded from the analysis.

b All estimates were adjusted for survey design and sample weights.

c Includes amidoarone, carbamazepine, chlorpropamide, carbidopa/levodopa, heparin, interferon, lithium, phenytoin, phenobarbital, or sulfasalazine.

* Significantly different by sex, *p* < 0.05.

**Table 4:** Associations [β (95% CI)] of ∑TEQs and ∑PCBs with thyroid hormones in women without thyroid disease.

	Association of total T_4_ with			Association of Ln TSH with		
Subgroup, cycle	Ln ∑PCBs	Ln ∑TEQs	Ln DDE	Ln ∑PCBs	Ln ∑TEQs	Ln DDE
All women						
1999–2000	−0.20 (−0.47 to 0.07) *n* = 333	−0.19 (−0.70 to 0.33) *n* = 310	0.16^a^ (−0.04 to 0.37) *n* = 350	−0.03 (−0.30 to 0.25) *n* = 332	0.15 (−0.14 to 0.44) *n* = 309	−0.01 (−0.12 to 0.11) *n* = 350
2001–2002	0.09 (−0.42 to 0.59) *n* = 476	−0.58* (−1.26 to 0.10) *n* = 386	0.11 (−0.07 to 0.30) *n* = 490	0.01 (−0.17 to 0.19) *n* = 475	0.06 (−0.15 to 0.27) *n* = 385	0.08 (−0.03 to 0.19) *n* = 489
Women < 60 years of age						
1999–2000	−0.08 (−0.40 to 0.25) *n* = 215	−0.04 (−0.78 to 0.69) *n* = 197	0.33^a^,** (0.04 to 0.62) *n* = 219	−0.04 (−0.36 to 0.28) *n* = 214	0.16 (−0.14 to 0.47) *n* = 196	−0.04 (−0.16 to 0.08) *n* = 219
2001–2002	0.20 (−0.35 to 0.76) *n* = 327	−0.51 (−1.30 to 0.29) *n* = 260	0.08 (−0.14 to 0.29) *n* = 337	−0.01 (−0.21 to 0.19) *n* = 326	0.04 (−0.27 to 0.35) *n* = 259	0.09 (−0.05 to 0.22) *n* = 336
Women > 60 years of age						
1999–2000	−0.38 (−0.89 to 0.14) *n* = 118	−0.40** (−0.71 to −0.10) *n* = 113	−0.47^a^,# (−0.74 to −0.20) *n* = 131	0.14 (−0.17 to 0.45) *n* = 118	0.00 (−0.48 to 0.48) *n* = 113	0.15^a^* (−0.01 to 0.30) *n* = 131
2001–2002	−0.96^a^,# (−1.51 to −0.41) *n* = 149	−1.20^a^,# (−1.75 to −0.64) *n* = 126	0.26^a^,* (−0.03 to 0.55) *n* = 153	0.25** (0.05 to 0.46) *n* = 149	0.23** (0.04 to 0.42) *n* = 126	0.05 (−0.04 to 0.15) *n* = 153

Value for the effect of ∑PCBs, ∑TEQs, or DDE individually on thyroid hormone is from the linear regression model adjusted for survey design and sample weights, total serum lipids, BMI, race, age, log serum cotinine, menopausal status, and medication use (furosamide, NSAIDs, beta-blockers, blood glucose regulators, and other medications). Effects of organochlorines on thyroid hormones were also estimated in linear regressions that simultaneously modeled for concentrations of ∑PCBs, ∑DDE, and TEQs, and the significance of the effects but not the beta coefficients are shown.

a For model including all three organochlorines, *p* < 0.05.

* 0.05 < *p* < 0.1,

** 0.01 < *p* < 0.05, and

# *p* < 0.01 for model using individual organochlorine.

**Table 5:** Associations [β (95% CI)] of ∑TEQs and PCBs with thyroid hormones in men without thyroid disease.

	Association of total T_4_ with			Association of Ln TSH with		
Subgroup, cycle	Ln ∑PCBs	Ln ∑TEQs	Ln DDE	Ln ∑PCBs	Ln ∑TEQs	Ln DDE
All men						
1999–2000	0.12 (−0.30 to 0.55) *n* = 436	−0.12 (−0.61 to 0.37) *n* = 402	−0.08 (−0.35 to 0.19) *n* = 454	−0.17 (−0.45 to 0.11) *n* = 436	−0.09 (−0.38 to 0.20) *n* = 402	−0.05 (−0.11 to 0.01) *n* = 454
2001–2002	−0.31 (−0.76 to 0.15) *n* = 653	−0.47* (−0.97 to 0.04) *n* = 497	−0.03 (−0.18 to 0.24) *n* = 667	−0.09 (−0.21 to 0.04) *n* = 653	−0.02 (−0.20 to 0.16) *n* = 497	0.04 (−0.03 to 0.10) *n* = 667
Men < 60 years of age						
1999–2000	−0.06 (−0.70 to 0.57) *n* = 278	−0.27 (−0.79 to 0.26) *n* = 252	−0.10 (−0.39 to 0.18) *n* = 286	−0.15 (−0.54 to 0.24) *n* = 278	−0.05 (−0.39 to 0.29) *n* = 252	−0.04 (−0.11 to 0.03) *n* = 286
2001–2002	−0.41 (−0.92 to 0.10) *n* = 467	−0.40 (−1.05 to 0.25) *n* = 342	−0.02 (−0.26 to 0.22) *n* = 472	−0.09 (−0.24 to 0.06) *n* = 467	−0.12 (−0.38 to 0.14) *n* = 342	0.02 (−0.05 to 0.09) *n* = 472
Men > 60 years of age						
1999–2000	0.19 (−0.36 to 0.74) *n* = 158	0.25 (−0.36 to 0.86) *n* = 150	−0.18^a^ (−0.47 to 0.11) *n* = 168	−0.19** (−0.38 to 0.00) *n* = 158	−0.22 (−0.54 to 0.10) *n* = 150	−0.09 (−0.25 to 0.08) *n* = 168
2001–2002	0.10 (−0.61 to 0.81) *n* = 186	−0.57^a^,* (−1.17 to 0.32) *n* = 155	0.21 (−0.19 to 0.60) *n* = 195	−0.18^a^,* (−0.37 to 0.01) *n* = 186	0.19 (−0.11 to 0.49) *n* = 155	0.10 (−0.06 to 0.26) *n* = 195

Value for the effect of ∑PCBs, ∑TEQs, or DDE individually on thyroid hormone is from the linear regression model adjusted for survey design and sample weights, total serum lipids, BMI, race, age, log serum cotinine, and medication use (furosamide, NSAIDs, beta-blockers, blood glucose regulators, and other medications). Effects of organochlorines on thyroid hormones were also estimated in linear regressions that simultaneously modeled for concentrations of ∑PCBs, ∑DDE, and TEQs, and the significance of the effects but not the beta coefficients are shown.

a For model including all three organochlorines, *p* < 0.05.

* 0.05 < *p* < 0.1, and

** *p* < 0.05 for model using individual organochlorine.

## References

[b1-ehp0115-001197] Bloom MS, Weiner JM, Vena JE, Beehler GP (2003). Exploring associations between serum levels of select organochlorines and thyroxine in a sample of New York State sportsmen: the New York State Angler Cohort Study. Environ Res.

[b2-ehp0115-001197] Blount BC, Pirkle JL, Osterloh JD, Valentine-Blasini L, Caldwell KL (2006). Urinary perchlorate and thyroid hormone levels in adolescent and adult men and women living in the United States. Environ Health Perspect.

[b3-ehp0115-001197] Brouwer A, Klasson-Wehler E, Bokdam M, Morse DC, Traag WA (1990). Competitive inhibition of thyroxin binding to transthyretin by monohydroxy metabolites of 3,4,3′,4′-tetra-chlorobiphenyl. Chemosphere.

[b4-ehp0115-001197] Calvert GM, Sweeney MH, Deddens J, Wall DK (1999). Evaluation of diabetes mellitus, serum glucose, and thyroid function among United States workers exposed to 2,3,7,8-tetra-chlorodibenzo-*p*-dioxin. Occup Environ Med.

[b5-ehp0115-001197] CDC (2005). Third National Report on Human Exposure to Environmental Chemicals.

[b6-ehp0115-001197] CDC (Centers for Disease Control and Prevention) (2007a). National Health Nutrition and Examination Survey.

[b7-ehp0115-001197] CDC (Centers for Disease Control and Prevention) (2007b). Laboratory Procedure Manual: PCBs and Persistent Pesticides.

[b8-ehp0115-001197] CDC (Centers for Disease Control and Prevention) (2007c). Laboratory Procedure Manual: PCDDS, PCDFs and cPCBs.

[b9-ehp0115-001197] CDC (Centers for Disease Control and Prevention) (2007d). Laboratory Procedure Manual: Thyroid Stimulating Hormone.

[b10-ehp0115-001197] CDC (Centers for Disease Control and Prevention) (2007e). Laboratory Procedure Manual: Thyroxin (T4).

[b11-ehp0115-001197] Fisher JW, Campbell J, Muralidhara S, Bruckner JV, Ferguson D, Mumtaz M (2006). Effect of PCB 126 on hepatic metabolism of thyroxine and perturbations in the hypothalamic-pituitary-thyroid axis in the rat. Toxicol Sci.

[b12-ehp0115-001197] Geyer HJ, Schramm KW, Feicht EA, Behechti A, Steinberg C, Bruggemann R (2002). Half-lives of tetra-, penta-, hexa-, hepta-, and octachlorodibenzo-*p*-dioxin in rats, monkeys, and humans – a critical review. Chemosphere.

[b13-ehp0115-001197] Hagmar L, Bjork J, Sjodin A, Bergman A, Erfurth EM (2001a). Plasma levels of persistent organohalogens and hormone levels in adult male humans. Arch Environ Health.

[b14-ehp0115-001197] Hagmar L, Rylander L, Dyremark E, Klasson-Wehler E, Erfurth EM (2001b). Plasma concentrations of persistent organochlorines in relation to thyrotropin and thyroid hormone levels in women. Int Arch Occup Environ Health.

[b15-ehp0115-001197] Hagmar L, Wallin E, Vessby B, Jonsson BA, Bergman A, Rylander L (2006). Intra-individual variations and time trends 1991–2001 in human serum levels of PCB, DDE and hexachlorobenzene. Chemosphere.

[b16-ehp0115-001197] Khan MA, Lichtensteiger CA, Faroon O, Mumtaz M, Schaeffer DJ, Hansen LG (2002). The hypothalamo-pituitary-thyroid (HPT) axis: a target of nonpersistent *ortho*-substituted PCB congeners. Toxicol Sci.

[b17-ehp0115-001197] Koopman-Esseboom C, Morse DC, Weisglas-Kuperus N, Lutkeschipholt IJ, Van der Paauw CG, Tuinstra LG (1994). Effects of dioxins and polychlorinated biphenyls on thyroid hormone status of pregnant women and their infants. Pediatr Res.

[b18-ehp0115-001197] Landi MT, Bertazzi PA, Baccarelli A, Consonni D, Masten S, Lucier G (2003). TCDD-mediated alterations in the AhR-dependent pathway in Seveso, Italy, 20 years after the accident. Carcinogenesis.

[b19-ehp0115-001197] Langer P, Kocan A, Tajtakova M, Petrik J, Chovancova J, Drobna B (2007). Fish from industrially polluted freshwater as the main source of organochlorinated pollutants and increased frequency of thyroid disorders and dysglycemia. Chemosphere.

[b20-ehp0115-001197] Langer P, Tajtakova M, Fodor G, Kocan A, Bohov P, Michalek J (1998). Increased thyroid volume and prevalence of thyroid disorders in an area heavily polluted by polychlorinated biphenyls. Eur J Endocrinol.

[b21-ehp0115-001197] Langer P, Tajtakova M, Kocan A, Petrik J, Koska J, Ksinatova L (2004). Preliminary fundamental aspects on the thyroid volume and function in the population of long term heavily polluted area in East Slovakia. Organohalogen Compounds.

[b22-ehp0115-001197] Meeker JD, Altshul L, Hauser R (2007). Serum PCBs, *p,p*′-DDE, and HCB predict thyroid hormone levels in men. Environ Res.

[b23-ehp0115-001197] Meier CA, Burger AC, Braerman LE, Utiger RD (2005). Effects of drugs and other substances on thyroid hormone synthesis and metabolism. Warner and Ingbar’s The Thyroid.

[b24-ehp0115-001197] Murai K, Okamura K, Tsuji H, Kajiwara E, Watanabe D, Akagi K (1987). Thyroid function in “Yusho” patients exposed to polychlorinated biphenyls (PCB). Environ Res.

[b25-ehp0115-001197] Nagayama J, Tsuji H, Iida T, Hirakawa H, Matsueda T, Ohki M (2001). Effects of contamination level of dioxins and related chemicals on thyroid hormones and immune response systems in patients with “Yusho”. Chemosphere.

[b26-ehp0115-001197] Needham LL, Barr DB, Caudill SP, Pirkle JL, Turner WE, Osterloh J (2005). Concentrations of environmental chemicals associated with neurodevelopmental effects in U.S. population. Neurotoxicol.

[b27-ehp0115-001197] Osius N, Karmaus W, Kruse H, Witten J (1999). Exposure to polychlorinated biphenyls and levels of thyroid hormones in children. Environ Health Perspect.

[b28-ehp0115-001197] Ott MG, Zober A, Germann C (1994). Laboratory results for selected target organs in 138 individuals occupationally exposed to TCDD. Chemosphere.

[b29-ehp0115-001197] Patterson DG, Canady R, Wong LY, Lee R, Turner WE, Caudill D (2004). Age specific dioxin TEQ reference range. Organohalogen Compounds.

[b30-ehp0115-001197] Pavuk M, Schecter AJ, Akhtar FZ, Michalek JE (2003). Serum 2,3,7,8-tetrachlorodibenzo-*p*-dioxin (TCDD) levels and thyroid function in Air Force veterans of the Vietnam War. Ann Epidemiol.

[b31-ehp0115-001197] Persky V, McCann K, Mallin K, Freels S, Piorkowski J, Chary LK (2002). The La Salle Electrical Utilities Company Morbidity Study I. ATSDR Monograph PB02-100121.

[b32-ehp0115-001197] Persky V, Turyk M, Anderson HA, Hanrahan LP, Falk C, Steenport DN (2001). The effects of PCB exposure and fish consumption on endogenous hormones. Environ Health Perspect.

[b33-ehp0115-001197] Rylander L, Wallin E, Jönssson BA, Stridsberg M, Erfurth EM, Hagmar L (2006). Associations between CB-153 and *p,p*′-DDE and hormone levels in serum in middle-aged and elderly men. Chemosphere.

[b34-ehp0115-001197] Sala M, Sunyer J, Herrero C, To-Figueras J, Grimalt J (2001). Association between serum concentrations of hexachlorobenzene and polychlorobiphenyls with thyroid hormone and liver enzymes in a sample of the general population. Occup Environ Med.

[b35-ehp0115-001197] Schecter A, Papke O, Tung KC, Joseph J, Harris TR, Dahlgren J (2005). Polybrominated diphenyl ether flame retardants in the U.S. population: current levels, temporal trends, and comparison with dioxins, dibenzofurans, and polychlorinated biphenyls. J Occup Environ Med.

[b36-ehp0115-001197] Schell LM, Gallo MV, DeCaprio AP, Hubicki L, Denham M, Ravenscroft J (2004). Thyroid function in relation to burden of PCBs, *p,p*′-DDE, HCB, mirex and lead among Akwesasne Mohawk youth: a preliminary study. Environ Toxicol Pharmacol.

[b37-ehp0115-001197] Schisterman EF, Whitcomb BW, Louis GM, Louis TA (2005). Lipid adjustment in the analysis of environmental contaminants and human health risks. Environ Health Perspect.

[b38-ehp0115-001197] StataCorp (2005). Stata Survey Data Reference Manual. Release 9.

[b39-ehp0115-001197] Takser L, Mergler D, Baldwin M, de Grosbois S, Smargiassi A, Lafond J (2005). Thyroid hormones in pregnancy in relation to environmental exposure to organochlorine compounds and mercury. Environ Health Perspect.

[b40-ehp0115-001197] Triebig G, Werle E, Päpke O, Heim G, Broding C, Ludwig H (1998). Effects of dioxins and furans on liver enzymes, lipid parameters, and thyroid hormones in former thermal metal recycling workers. Environ Health Perspect.

[b41-ehp0115-001197] Turyk ME, Anderson HA, Freels S, Chatterton R, Needham LL, Patterson DG (2006a). Associations of organochlorines with endogenous hormones in male Great Lakes fish consumers and nonconsumers. Environ Res.

[b42-ehp0115-001197] Turyk M, Anderson HA, Hanrahan LP, Falk C, Steenport DN, Needham LL (2006b). Relationship of serum levels of individual PCB, dioxin, and furan congeners, and DDE with Great Lakes sport-caught fish consumption. Environ Res.

[b43-ehp0115-001197] van Birgelen AP, Smit EA, Kampen IM, Groeneveld CN, Fase KM, Van der Kolk J (1995). Subchronic effects of 2,3,7,8-TCDD or PCBs on thyroid hormone metabolism: use in risk assessment. Eur J Pharmacol.

[b44-ehp0115-001197] van Birgelen APJM, van der Kolk J, Poiger H, van den Berg M, Brouwer A (1992). Interactive effects of 2,2′,4,4′,5,5′-hexa-cholorbiphenyl and 2,3,7,8-tetra-chlorodibenzo-*p*-dioxin on thyroid hormone, vitamin A, and vitamin K metabolism in the rat. Chemosphere.

[b45-ehp0115-001197] Van den Berg M, Birnbaum LS, Denison M, De Vito M, Farland W, Feeley M (2006). The 2005 World Health Organization reevaluation of human and mammalian toxic equivalency factors for dioxins and dioxin-like compounds. Toxicol Sci.

[b46-ehp0115-001197] Vanderpump M, Braerman LE, Utiger RD (2005). The epidemiology of thyroid diseases. Warner and Ingbar’s The Thyroid.

[b47-ehp0115-001197] Wang SL, Su PH, Jong SB, Guo YL, Chou WL, Päpke O (2005). In utero exposure to dioxins and polychlorinated biphenyls and its relations to thyroid function and growth hormone in newborns. Environ Health Perspect.

